# SEOM guideline in ovarian cancer 2014

**DOI:** 10.1007/s12094-014-1229-z

**Published:** 2014-10-29

**Authors:** A. Gonzalez-Martín, I. Bover, J. M. Del Campo, A. Redondo, L. Vidal

**Affiliations:** MD Anderson Cancer Center, Madrid, Spain

**Keywords:** Ovarian cancer, Treatment guidelines, First line, Recurrent disease

## Abstract

Ovarian cancer is the leading cause of death due to gynecological cancer and the 5th cause of death for cancer in women in Europe. Optimal management of patients with ovarian cancer needs the participation of a well-trained multidisciplinary team. In the last few years, we have observed a significant improvement in the knowledge of the molecular biology of the different histotypes of ovarian cancer that will probably change our standard of care in the forthcoming years. In this Guideline, we summarize the most current evidence for the medical management of ovarian cancer.

## Introduction

Despite improvements in surgery and chemotherapy, ovarian cancer is still the leading cause of death for gynecological cancer [1]. Unfortunately, there are no methods for early detection or prevention applicable to the general population, and majority of patients will present with advanced disease. For this reason, the only way to improve the survival of patients with ovarian cancer is to offer the patient the best therapeutic alternatives delivered by well-trained and motivated multidisciplinary team. The aim of this guideline is to summarize the current evidence and to give evidence-based recommendations for clinical practice.

## Methodology

This Guideline is based in great part in the GEICO (Grupo Español de Investigación en Cáncer de Ovario) Guideline that was published in 2013 which has been revised and adapted to the SEOM Guideline format. To assign a level of evidence and a grade of recommendation to the different statements of this treatment guideline, it was decided to use the Infectious Diseases Society of America-US Public Health Service Grading System for Ranking Recommendations in Clinical Guidelines to determine the quality of evidence and strength of recommendation in each of the consensus recommendations (Table 1).

**Table 1:** Strength of recommendation and quality of evidence score

Category, grade	Definition
Strength of recommendation	
A	Good evidence to support a recommendation for use
B	Moderate evidence to support a recommendation for use
C	Poor evidence to support a recommendation
D	Moderate evidence to support a recommendation against use
E	Good evidence to support a recommendation against use
Quality of evidence	
I	Evidence from ≥1 properly randomized, controlled trial
II	Evidence from ≥1 well-designed clinical trial, without randomization; from cohort or case-controlled analytic studies (preferably from >1 center); from multiple time series; or from dramatic results from uncontrolled experiments
III	Evidence from opinions of respected authorities, based on clinical experience, descriptive studies, or reports of expert committees

## Pathology and molecular diagnosis

Epithelial ovarian cancer is no longer considered a single disease, as it is composed of a diverse group of tumors that can be classified based on distinctive morphologic and molecular features [2]. Although we have not yet different therapeutic approaches for the different subtypes, they use to show a different natural behavior and prognosis. Nevertheless, there is an increasing number of clinical trials focused on specific subtypes (i.e. low-grade serous ovarian cancer or clear cell carcinoma) or patients with specific molecular alterations (i.e. patients with BRCA or PI3K mutations). Due to the above mentioned reasons, pathological classification of patients with epithelial ovarian cancer according the WHO recommendations is mandatory nowadays. Specific immune-histochemistry assays must be performed when required in the differential diagnosis of a particular patient (II, A). Table 2 summarizes the different subtypes and molecular features.

**Table 2:** Histological subtypes and markers

Subtype	CK-7	CK-20	WT-1	P53	RE	RP	B-catenina	Other/comments
High grade serous	+	−	+	+	±	±		BRCA germline-mutated (20 %) Transitional is classified as high-grad e serous with transitional features
Mucinous	+	±	∓		−	−		CDX2 variable PAX8 (50 %)+
Endometrioid	+	−	∓	∓	±	±	+(40–50 %)	
Clear cell	+	−	−		−	−		
Low-grade serous	+	−	+		±	±		MD Anderson two-tier grading system required

## Surgical treatment

Surgery is the cornerstone in staging and treatment of ovarian cancer. Based on published improved outcomes, it is recommended that a gynecologic oncologist surgeon perform the primary surgery [II, A] [1].

### Early disease (clinical stage I/II)

The aim is proper staging of disease and removal of all macroscopic tumors. Surgery can be performed either by laparotomy, which is the most accepted procedure, or minimally invasive surgery in selected patients if performed by an experienced gynecologic oncologist [II, A]. Procedures must comprise: thorough inspection and palpation of all peritoneal surface, total hysterectomy and bilateral salpingo-oophorectomy (TH + BSO), omentectomy, pelvic and bilateral aortic lymphadenectomy up to the renal vessels, biopsies of pelvic peritoneum, paracolic gutters and right infra diaphragmatic area, sampling of ascites or peritoneal washing for cytology (when no ascites is found) [3]. Appendectomy is recommended in mucinous tumors. Under-staged patients in previous surgery should be re-staged according to the same surgical principles mentioned above. For a young patient (<40 years) who wishes to maintain fertility, a unilateral salpingo-oophorectomy may be adequate for selected stage I tumors (Ia and Ic due to intraoperative rupture but with negative cytology, grade 1 or 2, but not stage Ib) in addition to the staging procedure. After fulfilling their gestational desire, salpingo-oophorectomy and hysterectomy are recommended [III, B] [4].

### Advanced disease (III–IV)

The standard treatment of advanced OC is cytoreductive surgery followed by platinum-based combination chemotherapy. Although the ultimate goal is cytoreduction to microscopic disease by removing all visible disease [5, 6], successful cytoreduction to small-volume disease (<1 cm) increases the frequency of complete response and overall survival [II, A]. According to the 4th Ovarian Cancer Consensus Conference held in Vancouver, the term “optimal” cytoreduction should be reserved for those with no macroscopic residual disease [1]. Some contraindications for the outcome of this “maximum” effort surgery have been pointed out such as the following: poor performance status, mesentery root involvement, extra-abdominal visceral disease, multiple intraparenchymal liver metastases, or intestinal massive-serosal carcinomatosis [II, A].

## Systemic therapy in first line

### Early stages

Adjuvant platinum-based chemotherapy after surgery is indicated in high-risk early stages (IA and IB Grade 3, clear cell tumors and any grade of stages IC and IIA) [I, A] [7]. However, there is no consensus on the need to treat stages IA/B Grade 2. Only low-risk patients (stages IA/B Grade I) with correct surgical staging require observation exclusively. The recommended regimen consists of at least 3 cycles of paclitaxel-carboplatin [I, A] [8].

### Advanced stages

#### Conventional chemotherapy

According to the 4th Ovarian Cancer Consensus Conference the standard treatment should include paclitaxel (175 mg/m^2^) and carboplatin (AUC 5–7.5) every 3 weeks for six cycles [I, A] [1]. For patients not eligible to receive a taxane (specifically paclitaxel), the combination of carboplatin and pegylated liposomal doxorubicin (PLD) could be considered an alternative option [9].

### Neoadjuvant chemotherapy (NAC)

The EORTC-55971 trial showed that in women with stage IIIC or IV ovarian cancer, primary debulking surgery followed by at least six cycles of platinum-based chemotherapy or three cycles of platinum-based NAC, followed by interval debulking surgery, and then at least three more cycles of platinum-based chemotherapy, achieved the same OS (29 months PDS vs 30 months NAC) [10]. However, some concerns have risen from the quality of the surgery performed in this trial and the wide use of NAC even in patients that would be candidate for optimal upfront debulking surgery [11]. In conclusion, NAC should be reserved for those who cannot tolerate PDS and/or for whom optimal cytoreduction is not feasible after an adequate evaluation performed by a surgical team well trained on cytoreduction [I, B].

#### Dose-dense regimen

A Japanese study evaluated the weekly (dose dense) administration of paclitaxel in combination with carboplatin every 3 weeks in patients with advanced ovarian cancer showing a benefit not only in PFS but also in OS [12]. However, the MITO-7 trial has not confirmed the dose-dense hypothesis with carboplatin and paclitaxel administered in a weekly schedule. For this reason, at least in Caucasian population dose dense cannot be consistently recommended [I, B], although it could be an option according to the 4th Ovarian Cancer Consensus Conference.

#### Intraperitoneal chemotherapy (IP CT)

Three large randomized studies and one meta-analysis have found improvements in PFS and OS when part of the chemotherapy is administered directly in the peritoneal cavity (Table 3) but with a significant increment in toxicity [13–16]. Nevertheless, IP CT is shown to be unmistakably superior to IV CT and is another standard option in the management of patients with stage III and residual tumor ≤1 cm after upfront surgery [I, A].

**Table 3:** Front line intraperitoneal studies

Study	Control regimen	Experimental regimen	Eligible patients	No. of patients	PFS IV/IP	OS IV/IP
SWOG 8501/GOG 104, Alberts et al. [13]	Cisplatin, 100 mg/m^2^ i.v.; cyclophosphamide, 600 mg/m^2^ i.v.; q 3 weeks × 6	Cisplatin, 100 mg/m^2^ i.p.; cyclophosphamide, 600 mg/m^2^ i.v.; q 3 weeks × 6	Stage III, ≤2 cm residual	546	–	41
					–	49
						*P* = 0.02
GOG 114/SWOG 9227, Markman et al. [14]	Cisplatin, 75 mg/m^2^ i.v.; paclitaxel, 135 mg/m^2^ 24-h i.v.; q 3 weeks × 6	Carboplatin, AUC 9 i.v.; q 28 days × 2; cisplatin, 100 mg/m^2^ i.p.; paclitaxel, 135 mg/m^2^ 24-h i.v.; q 3 weeks × 6	Stage III, ≤1 cm residual	462	22.5	52.5
					27.6	63.2
					*P* = 0.01	*P* = 0.05
GOG 172, Armstrong et al. [15]	Cisplatin, 75 mg/m^2^ i.v.; paclitaxel, 135 mg/m^2^ 24-h i.v.; q 3 weeks × 6	Paclitaxel, 135 mg/m^2^ 24-h i.v.; Cisplatin, 100 mg/m^2^ i.p.; paclitaxel, 60 mg/m^2^ i.p. on day 8; q 3 weeks × 6	Stage III, ≤1 cm residual	415	18.3	49.5
					23.8	66.9
					*P* = 0.05	*P* = 0.03

#### Antiangiogenic therapy

Based on the available data coming from 2 phase III trials (GOG-218, ICON-7), bevacizumab added to initial chemotherapy followed by a maintenance period should be deserved for patients who, following standard surgery, are found to have macroscopic residual disease [I, A] [17, 18]. According to exploratory analysis, the benefit seems to be more significant in patients with either stage III disease and residual disease >1 cm, or stage IV disease.

## Therapy for relapsed ovarian cancer

Approximately 70–80 % of patients diagnosed with EOC will suffer a relapse after receiving first-line chemotherapy based on platinum and taxane. Secondary cytoreduction may be appropriate in selected patients despite there is no level 1 evidence which demonstrates a survival advantage. [II, B].

However, for the majority of patients with recurrent EOC the treatment is based only on systemic therapy. There is no survival benefit in the treatment of recurrent OC with chemotherapy based exclusively on a rise in the CA 125, and it anticipates a deterioration in the quality of life [I, A] [19].

Relapsed patients are classified according to progression-free interval (PFI) in the following groups:

- Progression while receiving last line of platinum-based therapy or within 4 weeks of last platinum dose.
- Progression-free interval since last line of platinum of <6 months.
- Progression-free interval since last line of platinum of 6–12 months.
- Progression-free interval since last line of platinum of >12 months.

### Treatment of distinct subgroups defined by progression-free interval

#### Treatment of patients with a PFI <6 months

In these patients non-platinum single-agent therapy with paclitaxel, pegylated liposomal doxorubicin (PLD), topotecan or gemcitabine is the best palliative option. These drugs have shown response rates (RR) less than 20 % and median overall survival (OS) of 9–12 months. [I, A]. The addition of bevacizumab to standard monotherapy provides statistically significant and clinically meaningful improvement in PFS and objective RR [20].

#### Treatment of patients with a PFI >12 months

Patients with recurrent disease and a progression-free interval over 12 months are considered fully platinum sensitive, as they use to respond to retreatment with a platinum-based regimen. We have strong evidence, summarized in the Table 4, showing that a platinum-based combination is associated to a longer PFS and also OS in comparison to single-agent platinum chemotherapy [I, A] [21–24]. As there is no combination that can be considered superior in terms of efficacy, the selection between the different options should be based on the toxicity profile of them. A randomized trial of bevacizumab combined with carboplatin-gemcitabine and controlled with placebo demonstrated a significant improvement in PFS without any impact in OS. This regimen can be considered one of the standard options in this population [I, A] [25].

**Table 4:** Randomized clinical trials in platinum-sensitive relapse

Study	*N*	Prior	6–12 Months* (%)	Treatment	PFS (m)	HR	95 % CI	OS
		Taxane (%)						
ICON 4 [21]	802	43	25	Carboplatin	9	0.76	0.66–0.89	24 m
				Carboplatin-Pac	12			29 m*
GEICO 9801 [22]	81	87	42	Carboplatin	8.4	0.54	0.32–0.92	17 m
				Carboplatin-Pac	12.2			–
AGO-EORTC [23]	356	70	40	Carboplatin	5.8	0.72	0.58–0.90	17.3 m*
				Carboplatin-Gem	8.6			18 m
CALYPSO [24]	973	35	99	Carboplatin-Pac	9.4	0.821	0.72–0.94	33 m
				Carboplatin-PLD	11.3			30.7 m
OCEANS [25]	484	100	42	Carboplatin-gemcitabine	8.4	0.48	0.38–0.60	35.2
				Carboplatin-gemcitabine-bevacizumab	12.4			33.3

6–12 Months*: rate of patients with a PFI of 6–12 months; * *P* < 0.05

#### Treatment of patients with a PFI 6–12 months

Patients relapsing between 6 to 12 months after the last platinum-based chemotherapy use to have lower response to platinum and shorter PFS and OS. For this reason, different strategies beyond carboplatin-based regimens are under investigation in this group of patients. One of these strategies is the use of a non-platinum based regimen. A subgroup analysis of a randomized trial comparing trabectedin-PLD with PLD showed that those patients with a PFI of 6–12 months at relapse obtained an increment in OS when treated with trabectedin-PLD [26]. Based on this result, this combination could be considered as an alternative to platinum-based regimen in this clinical setting [II, A].
